# Up-regulation of 15-lipoxygenase enzymes and products in functional and non-functional pituitary adenomas

**DOI:** 10.1186/s12944-019-1089-1

**Published:** 2019-07-09

**Authors:** Alaleh Bayat Barooni, Mohammad Ghorbani, Vahid Salimi, Alimohammad Alimohammadi, Mohammad E. Khamseh, Hamideh Akbari, Mehrnaz Imani, Mitra Nourbakhsh, Alireza Sheikhi, Farzad Izak Shirian, Maryam Ameri, Masoumeh Tavakoli-Yaraki

**Affiliations:** 1 Department of Biochemistry, School of Medicine, Iran University of Medical Sciences, Tehran, Iran; 2 Division of Vascular and Endovascular Neurosurgery, Firoozgar Hospital, Iran University of Medical Sciences, Tehran, Iran; 3 Department of Virology, School of Public Health, Tehran University of Medical Sciences, Tehran, Iran; 4 Iranian Legal Medicine Organizations, Tehran, Iran; 5 Endocrine Research Center, Institute of Endocrinology and Metabolism, Iran University of Medical Sciences (IUMS), Tehran, Iran; 6 Clinical Research Development Unit (CRDU), Sayad Shirazi Hospital, Golestan University of Medical Sciences, Gorgan, Iran; 7 Forensic Medicine Department, Faculty of Medicine, Iran University of Medical Sciences, Tehran, Iran

**Keywords:** Pituitary adenoma, 15-Lipoxygense, 15(S) HETE, 13(S) HODE

## Abstract

**Background:**

Pituitary adenoma accounts as a complex and multifactorial intracranial neoplasm with wide range of clinical symptoms which its underlying molecular mechanism has yet to be determined. The bioactive lipid mediators received attentions toward their contribution in cancer cell proliferation, progression and death. Amongst, 15-Lipoxygense (15-Lox) enzymes and products display appealing role in cancer pathogenesis which their possible effect in pituitary adenoma tumor genesis is perused in the current study.

**Methods:**

The 15-Lipoxygenses isoforms expression level was evaluated in tumor tissues of prevalent functional and non-functional pituitary adenomas and normal pituitary tissues via Real-Time PCR. The circulating levels of 15(S) HETE and 13(S) HODE as 15-Lox main products were assessed in serum of patients and healthy subjects using enzyme immunoassay kits.

**Results:**

Our results revealed that 15-Lox-1 and 15-Lox-2 expression levels were elevated in tumor tissues of pituitary adenomas comparing to normal pituitary tissues. The elevated levels of both isoforms were accompanied with 15(S) HETE and 13(S) HODE elevation in the serum of patients. The 15-Lox-1 expression and activity was higher in invasive tumors as well as tumors with bigger size indicating the possible pro-tumorigenic role of 15-Lox-1, more than 15-Lox-2 in pituitary adenomas. The diagnostic value of 15-Lipoxygense isoforms and products were considerable between patients and healthy groups.

**Conclusion:**

The possible involvement of 15-Lipoxygense pathway especially 15-Lox-1 in the regulation of pituitary tumor growth and progression may open up new molecular mechanism regarding pituitary adenoma pathogenesis and might shed light on its new therapeutic strategies.

## Introduction

15-lipoxygenase (15-Lox) as a non-heam iron binding dioxygenase belongs to the master family of poly unsaturated fatty acids (PUFA) di-oxygenating enzymes which their metabolites are involved in normal cellular function, growth and development [1]. 15-Lox exists in various organs with the main expression pattern in blood cells likewise reticulocyte, eosinophil, macrophage, neutrophil, dendritic cell also epithelial, endothelial cells and fibroblasts [2]. The *15-Lox* gene is located in chromosome 17 and two closely-related forms of enzymes exist with similar structural features, 15-lipoxygenase-1 (15-Lox-1) that mainly oxidases linoleic acid and produces 13-S-hydroxyoctadecadienoic acid (13(S)-HODE) while the second form, 15-lipoxygenase-2 (15-Lox-2) catalysis arachidonic acid di-oxygenation and leads to the 15-S-Hydroxyeicosatetraenoic acid (15(S)-HETE) production [3]. The 15-Lox isoforms are regulated via several mediators likewise cytokines, histone modifications, methylation and growth factors [3–6]. Multiple evidences revealed that 15-Lox mediators are involved in the regulation of inflammatory responses and immune system function. For instance, lipoxins and resolvins elucidate remarkable anti-inflammatory effects on different human cell types [7–10]. It was also reported that the anti-inflammatory effects of 15-Lox mediators such as 15(S) HETE and 13(S) HODE might be occurred through activation of peroxisome proliferator-activated receptor (PPAR) γ which is a nuclear receptor involving in down regulation of pro-inflammatory cytokines [11]. Notably, 15(S) HETE and 13(S) HODE can mediate suppression of matrix metalloproteinases (MMPs) as promoters of cancer via activation of PPARγ [12]. However it was reported that activation of MAP kinase pathway through 15-Lox-1 and its product, 13(S) HODE, leads to the PPARγ suppression and tumor growth in prostate cancer while the effect of 15-Lox-2 is opposite [13].

Recent evidences provided insights into the implication of 15-Lox enzymes and mediators in pathogenesis of multiple diseases including asthma [14], viral infection [15], arthritis [12], cardiovascular diseases [16], autoimmune diseases [17] and cancer [18]. Recently, 15-Lox pathway has attained remarkable interests for its involvement in different cancer cell growth, proliferation, death and development [19]. For instance, it was shown that 15-Lox-2 overexpressed in lung adenocarcinoma which was associated with cancer cell proliferation and migration [18]. While, down regulation of 15-Lox-1 has been observed in colon cancer and enzyme restoration induced cancer cell apoptosis and reduced tumor genesis [20]. Notably, induction of 15-Lox-1 expression was associated with lower expression of metastasis-associated antigen 1 (MTA1) which was under transcriptional regulation of NF-κB in colorectal cancer [21]. Interestingly, 15-Lox-1 suppression was accompanied with decrease in the level of ferroptotic cell death mediators in cancer cells also 15-Lox-1 activation resulted in apoptotic cell death via mitochondrial impairment and ROS elevation [19, 22]. While the over expression of 15-Lox-1 was reported in human prostate cancer with positive effect on cancer cell progression. Notably, insulin-like growth factor-1 (IGF-1) induced cancer cell growth in a 15-Lox-1-dependent manner since 13(S) HODE might regulate IGF-1 expression transcriptionally [23]. Apparently the exact role of 15-Lox pathway in tumor genesis is controversial and requires further elucidations. Among the intracranial neoplasms, pituitary adenomas are common tumors which impose a great burden of morbidity on patients worldwide [24]. The uncontrolled proliferation of anterior pituitary gland resulted in several types of tumors with diverse pathological symptoms including acromegaly (growth hormone over production) prolactinoma (prolactin over production) and cushing’s disease (cortisol over production) as the most prevalent functional pituitary adenoma (FPA) also the non-functional pituitary adenomas (NFPA) which lacks hormone excess [25]. Based on the critical role of pituitary gland in metabolism and human health overall, pituitary gland tumors might affect many physiological functions in human body and characterizing the mechanistic details underlying tumor initiation and progression will give rise to the more efficient disease managing approaches. Unraveling the possible involvement of 15-Lox pathway in pituitary adenoma pathogenesis might brighten the molecular mechanisms underlying tumor formation in pituitary gland and can be exploited further in tumor management strategies. Therefore, this study is aimed to investigate the 15-Lox isoforms expression level and their main products in the prevalent types of pituitary adenoma tumor and blood samples and delineate the correlation of 15-Lox mediators with patient’s clinic pathological properties.

## Material and method

### Patients and sample collection

The total number of 75 pituitary samples including 55 pituitary adenomas and 20 cadaveric healthy pituitary tissues were enrolled in the current study with local ethical approval and informed consent. The ethics committee of vice president of research in Iran University of Medical Sciences approved the project ethically. The pituitary adenoma tumor samples were obtained from patients with pituitary adenoma whom were subjected to the endoscopic transnasal transphenoidal surgery (ETSS) at the neurosurgery department of our institute. In order to compare the expression pattern of pathological and normal situation, the healthy pituitary tissues were collected from Legal Medicine Organization (LMO), Tehran, Iran at the first hour of their death. Following surgical resection, the pituitary fresh tumor tissues were divided into two sections, one transferred to the pathology department for further histological evaluations and the other part was taken away and kept in RNA latter (Qiagen, Germany) immediately and stored in − 80 °C until usage. Following tumor removal, the tissue histopatholoical characters were confirmed with expert pathologist. The autopsies of human normal pituitary tissues were collected from 20 patients with no pathological pituitary problems following the first hours of their death. A total amount of 6 ml venous blood samples were taken from all participants and collected in EDTA tubes and subjected to serum separation. As it is shown in Tables 1, 45.45% of patients with non-functional pituitary adenoma (NFPA) and 54.54% of patients with functional pituitary adenoma (FPA) were participated in this study. Among functional pituitary adenoma, 56.66% of patients had acromegaly and 43.33% of patients had Cushing. The clinic pathological features of patients are summarized in Table 1. Also the biochemical findings of all patients are presented in Table 2.

**Table 1:** The clinic- pathological features of patients with pituitary adenoma

Parameter	Groups	Pituitary adenoma (*n* = 55)	Non-Functional pituitary adenoma (*n* = 25)	Functional pituitary adenoma (*n* = 30)	Acromegaly (*n* = 17)	Cushing (*n* = 13)
Age (Years)	20–40	27 (49.09%)	11 (44%)	15 (50%)	9 (52.94%)	7 (53.84%)
	40–60	17 (30.9%)	7 (28%)	10 (33.33%)	5 (29.41%)	
	60≤	11 (20%)	7 (28%)	5 (16.66%)	3 (17.64%)	5 (38.46%)
						1 (7.69%)
Gender	Female	26 (47.27%)	9 (36%)	19 (63.33%)	9 (52.94%)	8 (61.53%)
	Male	29 (52.72%)	16 (64%)			
				11 (36.66%)	8 (47.05%)	5 (38.46%)
Tumor invasiveness	Invasive	25 (45.45%)	10 (40%)	13 (43.43%)	9 (52.94%)	4 (30.76%)
	Non- Invasive	30 (54.54%)	15 (60%)	17 (56.56%)	8 (47.05%)	9 (69.23%)
Tumor size	Micro-Adenoma	11 (20%)	0	13 (43.43%)	5 (29.41%)	8 (61.53%)
	Macro-Adenoma	44 (80%)	25 (100%)	17 (56.56%)	12 (70.58%)	5 (38.46%)

**Table 2:** The biochemical features of patients with pituitary adenoma

Demographic features	NFPA	Acromegaly	Cushing
Age	53.72 ± 4.082	47.88 ± 4.2	32.3 ± 3.3
Tumor size	28.23 ± 2.73	14.25 ± 1.43	9.87 ± 2.74
Blood Sugar (100-145 mg/dl)	153 ± 8.7	156.1 ± 13.2	211.3 ± 49.10
Na (135–145 mmol/L)	142.2 ± 1.6	142.4 ± 1.13	153.6 ± 4.5
K (3.5–5.5 mmol/L)	3.86 ± 1.16	4.04 ± 0.11	3.88 ± 0.26
Urea (14-43 mg/dl)	41.80 ± 7.88	39.48 ± 4.4	47.67 ± 13.2
WBC (4–10*1000/mm^3^)	9.6 ± 0.63	7.94 ± 0.51	11.13 ± 0.9
Prolactin	259.2 ± 140	691 ± 30	170 ± 96
Men: 87–392 mIU/L Women	651.7 ± 33	909 ± 61	319 ± 52
productive age: 132–498 mIU/			
Post menopause: 90–392 mIU/L			
Growth Hormone Women:0.126–9.88 ng/ml Men:0.03–2.47 ng/ml	0.17 ± 0.07 0.28 ± 0.07	14.71 ± 5.01 6.64 ± 2.3	0.09 ± 0.006 0.40 ± 0.26
ACTH (7.2–63.3 pg/ml)	24.4 ± 7.4	40.49 ± 7.18	215.1 ± 95.55
Cortisol (morning)6.2–19.4 μg/dl	12.59 ± 4.77	21.20 ± 5.5	30.63 ± 10.2
Testosterone Men:2.5–10 ng/ml women:0.2–0.95 ng/ml	3.3 ± 0.89 0.2 ± 0.02	1.17 ± 0.37 0.3 ± 0.1	1.86 ± 0.92 1.04 ± 0.7
IGF-1 53-234 ng/ml	187.6 ± 33.3	675.9 ± 68.62	226 ± 32

### RNA extraction, cDNA synthesis, real-time PCR

**Fig. 1:**
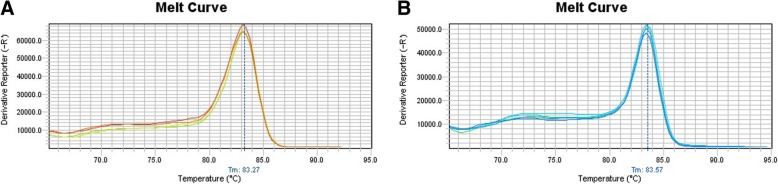
The Melt curve analysis of 15-lox-1 (**a**), 15-Lox-2 (**b**)

To evaluate the expression level of 15-Lox isoforms, the tumor and healthy pituitary tissues were applied for RNA extraction via Trizol (Invitrogen, Grand Island, USA) based on the manufacturer’s instruction. The extracted RNA from each tissue was evaluated as a point of quality and quantity with Nanodrop spectrophotometer (Nanodrop Technologies). The cDNA was synthesized from 1 μg of RNA via PrimeScript First Strand cDNA Synthesis Kit Takara, Japan) according to the manufacturer’s instruction. The Real-Time PCR using SYBR Premix Ex Taq II (Takara, Japan) was performed on the synthetized cDNA using Applied Biosystems Step One Plus, Real time system (Applied Biosystems, USA). The gene expression was assayed based on the running program as: 1 cycle at 95 °C for 5 min following 40 cycles at 95 °C for 5 S, 55 °C for 20 S and 60 °C for 35 S. The specificity of PCR products were confirmed by melting curve analysis for each amplified product (Fig. 1). As a housekeeping gene, the beta-actin expression level was measured to normalize the 15-Lox isoforms expression levels and the comparative CT (2^-ΔCt^) method was applied for analysis of gene expression. The designed primers which were used are listed in Table 3.

**Table 3:** Primers used for qRT-PCR assessment of gene expressions

Gene	Primers	Primer sequence	Tm
*15-lox-1*	Forward Reverse	5′ –CAC AGA GAT CCA GTT GCA GA −3 5′- GGC AAG GAG ACA GAA CTC AA-3’	58
*15-lox-2*	Forward Reverse	5′-GCC TTG AAC TTC TGA CCT CA- 3′ 5′-AGC ATC CAC TGA TTG GAC CT- 3’	58
Beta-Actin	Forward Reverse	5-GAT CTC CTT CTG CAT CCT GT-3′ 5′-TGG GCA TCC ACG AAA CTA C- 3’	57

### ELISA measurement of 15(S) HETE and 13(S) HODE level

The level of 15-Lox product namely 13(S) HODE was measured using an enzyme immunoassay kit (Abnova, Taiwan) based on the manufacturer’s protocol. The detection was performed at 450 nm via microplate reader. The minimum detectable range of 13(S) HODE was 1.6 ng/m. Also the level of 15(S) HETE was measured using an enzyme immunoassay kit (Abnova, Taiwan) according to the manufacturer’s instructions. The attachment of free 15(S) HETE to the polyclonal 15(S) HETE in a competitive manner leads to color generation which was detected at 405 nm via microplate reader. The minimum detectable range of 15(S) HETE was 69.21 pg/ml.

### Statistical analysis

For gene expression analysis, the comparative CT (2^-ΔCt^) method was applied also evaluating the correlations among patient’s clinic pathological features and gene expressions were delineated using the Pearson correlation coefficient test. Analysis of the target expression differences between groups was assessed via t-test. The receiver operating characteristic (ROC) curves and calculation of area under the curve (AUC) were performed to determine the 15-Lox isoforms and products diagnostic performance as possible pituitary adenoma biomarkers. The Graph Pad Prism version 6 (Graph Pad Software, San Diego California) and Statistical Package for Social Science (SPSS v.20) were used for calculation of all statistics. *P* values < 0.05 (two-sided) were considered statistically significant.

## Results

### The expression level of 15-Lox-1 in tumor tissues of different pituitary adenomas

**Fig. 2:**
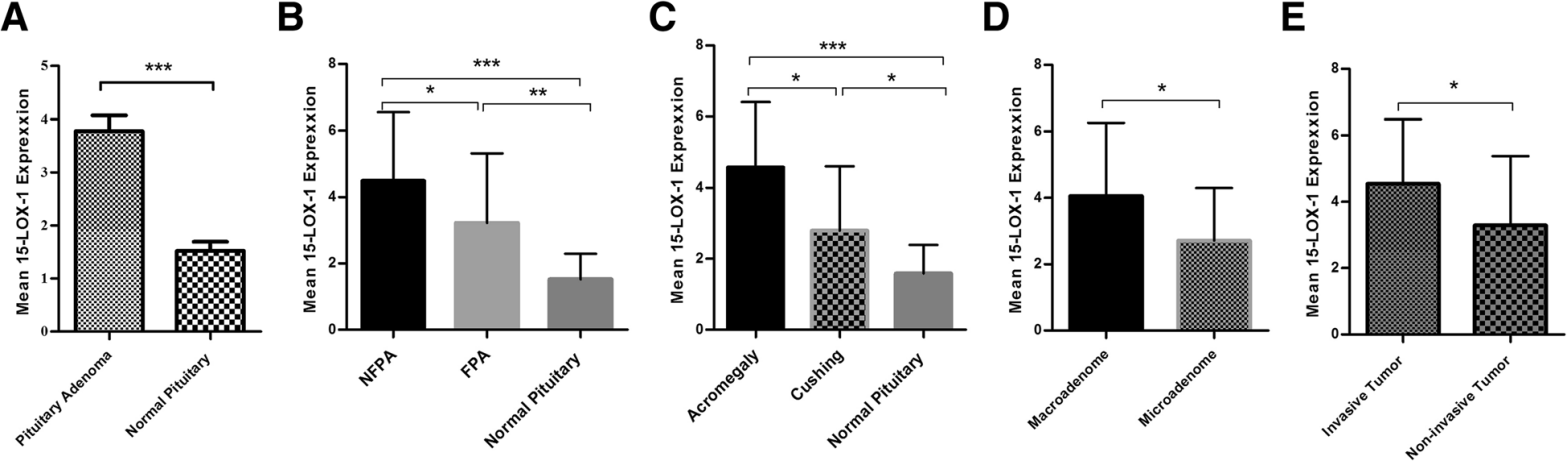
15-Lox-1 expression level in pituitary adenomas. The expression level of 15-Lox-1 was evaluated in tumor and normal tissues of pituitary with different adenoma types. The 15-Lox-1 expression level was increased in pituitary adenoma (**a**), non-functional pituitary adenoma (**b**), different types of functional pituitary adenoma (**c**), macroadenoma (**d**) and invasive (**e**) tumor tissues. The Statistical differences between groups is shown as asterisk (* = *P* < 0.05, ** = *P* < 0.01,*** = *P* < 0.001)

To delineate the role of 15-Lox-1 in pituitary adenoma pathogenesis, the 15-Lox-1 gene expression was assessed using Real-Time PCR in 55 pituitary adenoma tumors and 20 normal pituitary tissues. As it is shown in Fig. 2a, the 15-Lox-1 expression level was increased significantly in tumor tissues comparing to normal pituitary tissues (*P* = 0.0001). Based on analysis, the mean and standard error mean (SEM) of 15-Lox-1 mRNA level which was calculated via 2^-ΔCt^ method was 3.77 ± 0.28 and 1.52 ± 0.17 in patients and control groups, respectively. Notably, the approximate of 2.48 fold increase in the 15-Lox-1 expression was detected in tumor comparing to healthy pituitary tissues. Additionally, the expression level of 15-Lox-1 was elevated significantly in both FPA and NFPA comparing to normal pituitary also the NFPA group showed higher level of 15-Lox-1 expression in comparison to FPA (*P* = *0.027)* (Fig. 2b). The significant elevation of 15-Lox-1 expression level was also observed in acromegaly and Cushing tumor tissues comparing to normal pituitary (*P* = 0.001, *P* = 0.02, respectively) moreover 15-Lox-1 expressed more in acromegaly comparing to Cushing patients (*P* = 0.01) (Fig. 2c). The tumor tissues were evaluated as a matter of size and invasiveness and 45.45% of tumor tissues which were participated in our study were invasive and 54.54% of tumors were non-invasive. Also 80% of tumor tissues were categorized as macroadenoma and 20% were in the group of microadenoma. Our results showed higher expression of 15-Lox-1 in macroadenoma comparing to microdenoma (*P* = 0.05) (Fig. 2d). Also invasive tumor tissues revealed higher expression of 15-Lox-1 in comparison to non-invasive pituitary tumors (*P* = 0.02) (Fig. 2e). The correlation of patient’s clinic pathological features with 15-Lox-1 expression revealed no significant correlation within main features in different pituitary adenoma types which are summarized in Table 4.

**Table 4:** 15-Lox-1 Correlation with patient’s clinic pathological features

Parameter	Groups	Non-Functional pituitary adenoma *(P value)*	Acromegaly *(P value)*	Cushing *(P value)*
Age (Years)	20–40 40–60 60≤	0.20 0.35 0.38	0.09 0.98	0.74
Gender	Female Male	0.27	0.06	0.27
Tumor invasiveness	Invasive Non- Invasive	0.38	0.18	0.13
Tumor size	Micro-Adenoma Macro-adenoma	0	0.94	0.34

### The expression level of 15-Lox-2 in tumor tissues of different pituitary adenomas

**Fig. 3:**
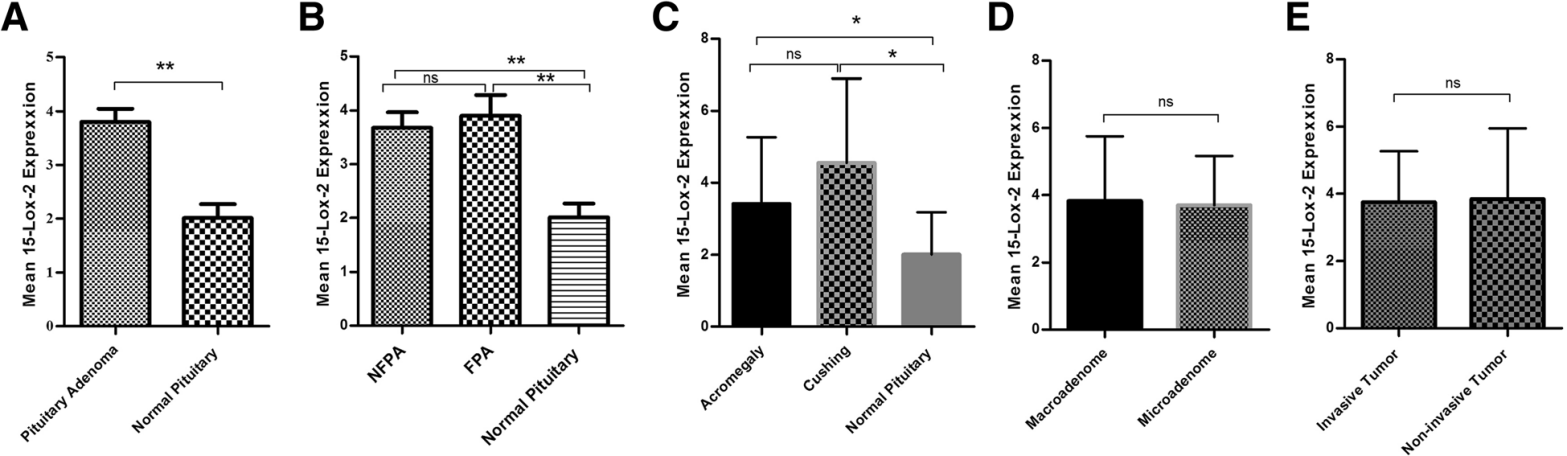
15-Lox-2 expression level in pituitary adenomas. The expression level of 15-Lox-2 was evaluated in tumor and normal tissues of pituitary with different adenoma types. The 15-Lox-2 expression level was increased in pituitary adenoma (**a**), non-functional pituitary adenoma (**b**), different types of functional pituitary adenoma (**c**), macroadenoma (**d**) and invasive (**e**) tumor tissues. The Statistical differences between groups is shown as asterisk (* = *P* < 0.05, ** = *P* < 0.01,*** = *P* < 0.001)

In order to evaluate the role of 15-Lox comprehensively, the expression level of 15-Lox-2 was assessed in tumor and normal tissues of pituitary. Our results revealed that the 15-Lox-2 expression level was elevated in tumor comparing to normal tissues significantly (*P* = 0.0014) (Fig. 3a). The analysis showed the mean and standard error means (SEM) of 15-Lox-2 mRNA level as 3.8 ± 0.24 and 2.00 ± 0.26 in patient and control groups, respectively which demonstrates the approximate of 1.9 fold increase in the 15-Lox-2 expression level in tumor tissues. By considering the expression level of 15-Lox-2 in FPA and NFPA (*P* = 0.001), our data showed a significant higher expression level in both groups in comparison to normal tissues although the difference between two groups was not remarkable (Fig. 3b). Among FPAs, both Cushing and acromegaly tumor tissues express more 15-Lox-2 comparing to normal tissues (*P* = 0.001) although the difference of expression between acromegaly and Cushing was not statistically significant (Fig. 3c). Based on our observation, the expression level of 15-Lox-2 showed no significant differences among macro and microadenoma (Fig. 3d) also invasive and non-invasive tumors (Fig. 3e). The correlation of patient’s clinic pathological features with 15-Lox-2 expression are summarized in Table 5 which shows no significant correlation in patient’s main features with 15-Lox-2 expression level.

**Table 5:** 15-Lox-2 Correlation with patient’s clinic pathological features

Parameter	Groups	Non-Functional pituitary adenoma *(P value)*	Acromegaly *(P value)*	Cushing *(P value)*
Age (Years)	20–40 40–60 60≤	0.47 0.68 0.89	0.78 0.47	0.64
Gender	Female Male	0.56	0.69	0.52
Tumor invasiveness	Invasive Non- Invasive	0.8	0.09	0.75
Tumor size	Micro-Adenoma Macro-adenoma	0	0.8	0.52

### 15(S) HETE and 13(S) HODE levels in pituitary adenomas and healthy subjects

**Fig. 4:**
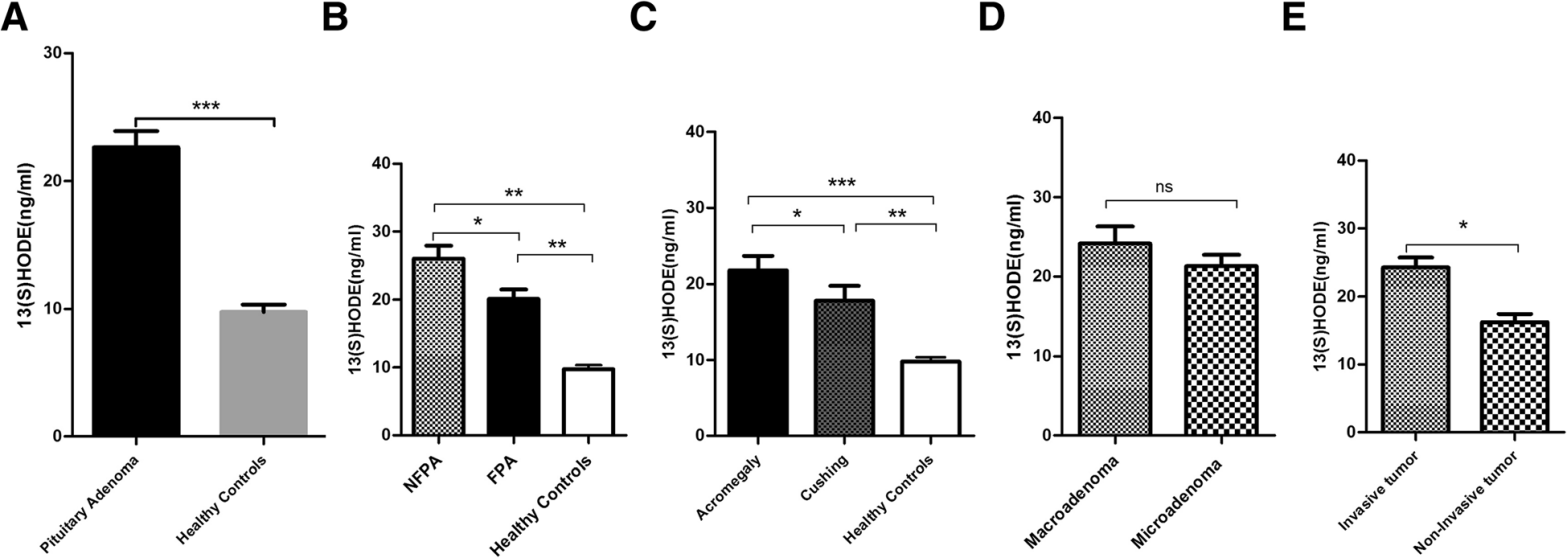
13(S) HODE level in pituitary adenomas. The level of 13(S) HODE was evaluated in patients with different types of pituitary adenomas and healthy controls (**a**). The level of 13(S) HODE in Patients with pituitary adenomas (B) according to the FPA and NFPA type of tumor (**b**), types of FPA (**c**), tumor size (**d**), invasion (**e**) was determined (* = *P* < 0.05, ** = *P* < 0.01,*** = *P* < 0.001)

**Fig. 5:**
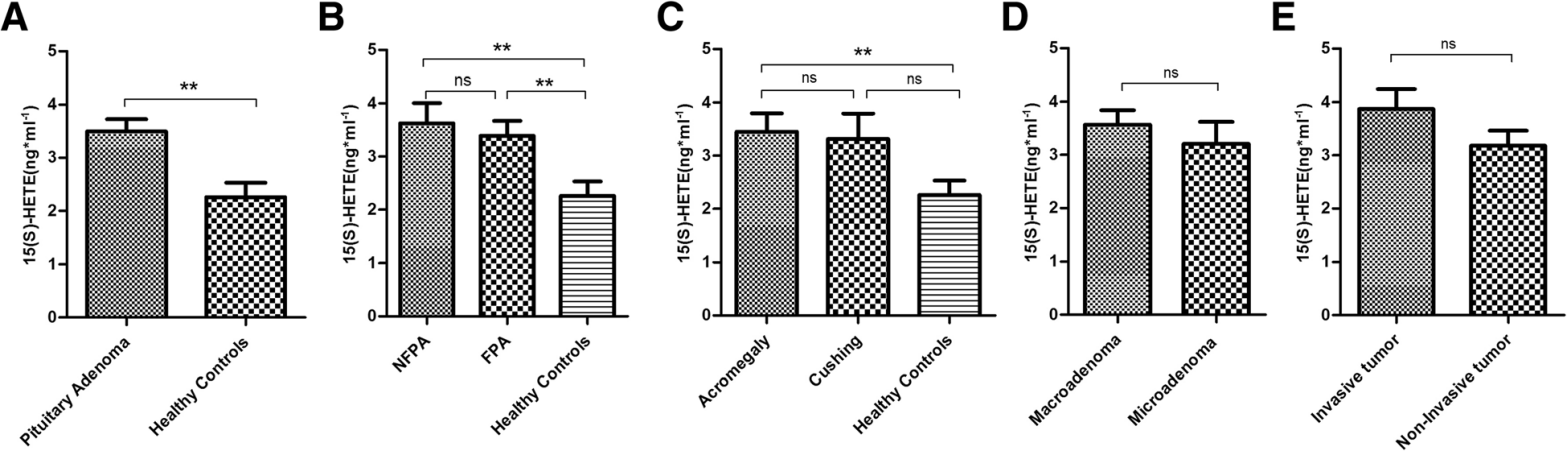
15(S) HETE level in pituitary adenomas. The level of 15(S) HETE was evaluated in patients with different types of pituitary adenomas and healthy controls (**a**). The level of 15(S) HETE in Patients with pituitary adenomas (B) according to the FPA and NFPA type of tumor (**b**), types of FPA (**c**), tumor size (**d**), invasion (**e**) was determined (* = *P* < 0.05, ** = *P* < 0.01,*** = *P* < 0.001)

In order to evaluate the 15-Lox-1 and 2 main products in pituitary adenoma pathogenesis, the level of 13(S) HODE and 15(S) HETE as 15-Lox-1 and 2 main products, respectively were assessed in serum of patients and healthy subjects. Based on our data, the serum level of 13(S) HODE was significantly increased in patients with pituitary adenoma comparing to healthy controls (*P* = 0.001) (Fig. 4a). Also considering the 13(S) HODE level in different patient groups, our data revealed higher level of 13(S) HODE in both FPA and NFPA comparing to healthy subjects (*P* = 0.001) meanwhile the differences between these groups were statistically significant (*P* = 0.03) (Fig. 4b). Notably, patients with acromegaly had higher level of 13(S) HODE comparing to Cushing type of pituitary adenoma (*P* = 0.05) also the level of 13(S) HODE was significantly higher in both groups comparing to normal subjects (Fig. 4c). As a matter of size and invasive features of tumors, our data showed a significant higher level of 13(S) HODE in serum of patients with invasive pituitary tumor comparing to non-invasive type of tumor (*P* = 0.01) (Fig. 4e) while the higher level of this product in patients with macroadenoma was not statistically significant comparing to microadenoma (Fig. 4d). The assessment of 15(S) HETE level in serum of patient and healthy groups showed a significant higher level in patients comparing to healthy subjects (*P* = 0.004) (Fig. 5a). Also, the higher level of 15(S) HETE was observed in both FPA and NFPA comparing to the healthy controls (*P* = 0.007, *P* = 0.004, respectively) while no significant difference was detected within these two groups (Fig. 5b). Based on the data, only patients with acromegaly showed a significant higher level of 15(S) HETE in serum comparing to healthy subjects (*P* = 0.004) however the level of this product in Cushing patients was not remarkable (Fig. 5c). Moreover, despite of higher 15(S) HETE level in patients with macroadenoma and invasive tumors, 15(S) HETE revealed no significant differences in patients with bigger size (Fig. 5d) and invasive tumors (Fig. 5e) comparing to microadenoma and non-invasive tumor.

### The diagnostic value of 15-lox isoforms and products in pituitary adenomas

**Fig. 6:**
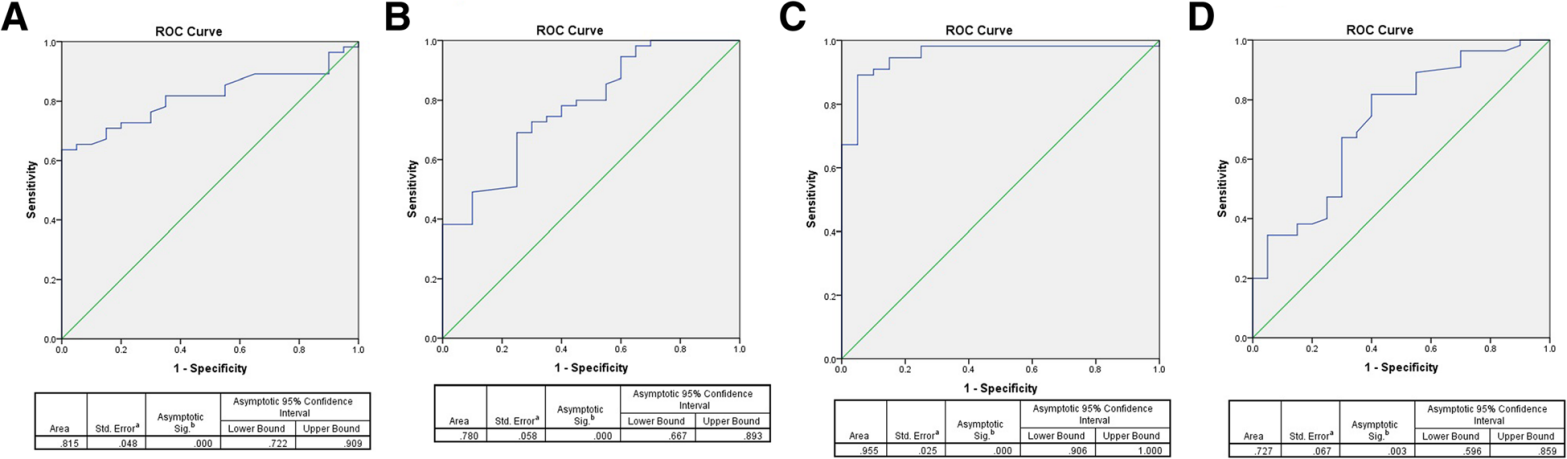
15-Lox isoforms and products ROC curve. The 15-Lox isoforms and products ROC Curve, area under the curve, 95% confidence interval, *P* value are shown for 15-Lox-1 (**a**), 15-Lox-2 (**b**), 13(S) HODE (**c**), 15(S) HETE (**d**)

The receiver operating characteristic (ROC) curve was further constructed to determine the possible diagnostic value of 15-Lox-1 isoforms and their products levels within patients with pituitary adenoma and healthy subjects. The area under the curve (AUC) and the optimal cutoff of mRNA expression level was determined subsequently for each tested group. As it is shown in Fig. 6a, the ROC curve of 15-Lox-1 expression in pituitary adenoma tumor and normal pituitary tissues reveled the AUC level of 0.815 (95% CI, 0.722–0.909, *P* = 0.0001) with the best cut off value of 1.72 which had 76 and 70% sensitivity and specificity, respectively based on the Youden Index. Additionally, the 15-Lox-2 expression demonstrated the AUC level of 0.78 (95% CI, 0.66–0.89, *P* = 0.0001) with the best cut off value of 2.67 which had 70 and 70% sensitivity and specificity respectively (Fig. 6b). Regarding the diagnostic value of 15-Lox products, our data showed the AUC level of 0.955 (95% CI, 0.90–1, *P* = 0.0001) with the best cut off value of 12.35 which had 94 and 80% sensitivity and specificity respectively for 13(S) HODE in patients and healthy subjects (Fig. 6c) however 15(S) HETE revealed the AUC level of 0.727 (95% CI, 0.596–0.859, *P* = 0.003) with the best cut off value of 2.34 which had 74% sensitivity and 60% specificity (Fig. 6d).

## Discussion

Pituitary adenomas account around 15% of intracranial neoplasms with the surgical resection as its most effective therapeutic approach and the tumor recurrence in considerable cases. Despite extensive efforts, the molecular mechanisms underlying pituitary tumor pathogenesis is uncertain and has yet to be determined [26]. Recently, the involvement of bioactive lipids and arachidonic acid-related mediators in regulating diseases outcomes likewise diabetes mellitus [27], autoimmune diseases [17], asthma [14] and aging [28] has been attended. Moreover, the aberrant lipid signaling and metabolism considered as a hallmark of different types of cancer cells. The cancer cell lipid composition and elevated lipid metabolites to providing energy for cell proliferation, in one hand and triggering intracellular inflammatory pathways via lipid mediators to facilitate cell invasion in another hand, make lipid-related enzymes and mediators appealing in cancer management. 15-Lox as a central enzyme in PUFA di-oxygenation, is involved in several cell functions likewise cell proliferation, development and inflammation that spotted this enzyme as a possible effector in cancer cell metabolism. In the present study the expression levels of 15-Lox enzyme isoforms as well as their main products were assessed in patients with most prevalent forms of pituitary adenomas. The elevated levels of both 15-Lox-1 and 2 expressions were observed in tumor tissues of patients with pituitary gland adenomas comparing to healthy tissues. No similar studies were found to evaluate the status of 15-Lox enzymes in human pituitary adenoma however it was shown that 15-HETE and 15-HPETE stimulated the GH3 rat pituitary adenoma cell line to produce pituitary hormones [29]. It was demonstrated that the exposure of LbetaT2 (gonadotrope cell line) cells to gonadotropin-releasing hormone enhanced the expression level of 12-Lox and 12-HETE as the enzyme main product [30]. Moreover, it was shown that inhibition of eicosanoid pathway enzymes such as cyclooxygenase leads to blockage of ACTH secretion following induction by IL-1beta although suppression of 5-Lox had no specific effect on the hypothalamic-pituitary-adrenal (HPA) axis hormone secretion [31]. In support of this, the arachidonic acid metabolites (15-HETE and 5-HETE) induced gonadotropin release in rat pituitary cells with the more potency which was observed for 15-HETE [32]. Based on our data, the higher level of 15-Lox-2 and its metabolite 15-HETE was observed in hormone-secreting pituitary tumors comparing to NFPAs which might support the previous evidences on the effective role of 15-HETE on inducing pituitary hormone release. The overexpression of 15-Lox-1 was reported in lung adenocarcinoma [18] and prostate neoplasms [23] while its lower expression was observed in colon [33], esophageal [34], pancreatic [35] and breast [36] cancers. The up-regulation of 15-Lox-1 and its product in pituitary adenomas is in favor of its pro-tumorigenic role and can emphasize on the idea that 15-Lox-1 might be expressed in a tissue-dependent manner. Based on our data, 15-Lox-1 highly expressed in GH-secreting pituitary adenoma which had the elevated level of IGF-1 as a consequence of the disease. Our data was in line with the study of Kelavkar et al., that revealed the relevance of 15-Lox-1 with IGF-1 and the elevated prostate cancer cell proliferation however the correlation of 15-Lox-1 and IGF-1 in acromegaly is required further mechanistic surveys [23]. According to our results, invasive pituitary adenoma exhibited higher level of 15-Lox-1 expression in comparison to non-invasive tumors which this observation is in line with the oncogenic role of 15-Lox-1 and its increased expression in tumors with higher grade [18]. Since the invasion of tumor cells to the lymphatic vessels and their ability to metastasis can be mediated through 15-Lox-1 in breast carcinoma [37]. The relevance of cellular pathways such as STAT3 with 15-Lox-regulated cancer cell proliferation as well as the activation of intracellular signaling pathways such as Ras, MAP kinase and NF-κB with 15-Lox products have been demonstrated previously [18, 38] and enlighten the correlation of 15-Lox-1 with cell proliferation since our data demonstrated the higher level of 15-Lox-1 in tumors with higher proliferation rate (macroadenomas). Based on our data, the expression level of 15-Lox-1 and 13(S) HODE was mainly higher in patients with non-functional form of pituitary adenoma comparing to the functional form which might emphasize on the accelerating role of 15-Lox in inducing higher rate of cell proliferation in NFPAs that have tumors with bigger size. As the clinical point of view, the bigger tumor size of NFPAs is rationally due to the accelerated cell proliferation and reduced cell apoptosis which facilitate tumor growth and invasion [39] . In support of this, it was shown that the apoptotic index was considerably lower in NFPAs comparing to the rest of pituitary adenomas likewise the level of p27 KIP1, the cyclin- dependent kinase inhibitor, responsible for inhibiting cell cycle was reduced in NFPAs and pituitary carcinomas. Subsequently the higher expression of 15-Lox-1 in NFPAs in one hand can be due to higher rate of cell proliferation in these tumors and on the other hand, can be due to the direct impact of 15-Lox-1 on cellular proliferation mediators although it requires further assessments. 15-Lox-2 as a human-specific lipid-peroxidizing enzyme is mainly expressed in epithelial cells and shares around 40% amino acid identity with 15-Lox-1 which has both anti and pro-tumorigenic features. Despite the fact that 15-Lox isoforms belong to the same family, they differ as a point of distribution and substrate preference thus their role in cancer progression might be similar or opposite in different tissues. Accordingly, 15-Lox-1 and 13(S) HODE were up regulated in prostate tumors while 15-Lox-2 and 15(S) HETE were significantly down-regulated in prostate tumor tissues [40]. Notably, it was reported that 15-Lox-1 and its product activate MAP kinase pathway and subsequently reduced the activity of PPARγ in prostate and colorectal carcinoma cells while 15-Lox-2 and 15(S) HETE suppress MAP kinase pathway [13]. In contrast, both 15-Lox isoforms were reduced in breast cancer tumor tissues in the same manner and paly tumor suppresser role [36]. Our data revealed the same pattern of expression for both 15-Lox isoforms in pituitary adenomas and the enhanced level of circulating 15-Lox products in the patients although 15-Lox-1 and 13(S) HODE had better diagnostic values in patients and control groups comparing to 15-Lox-2 and 15(S) HETE. Based on our data, both 15-Lox-1 and 15-Lox-2 isoforms clearly expressed more in pituitary tumor tissues comparing to normal tissues although the increasing level of 15-Lox-1 in macro adenomas, invasive and NFPAs tumor tissues were more obvious than 15-Lox-2. Based on our results the level of 15(S) HETE and 13(S) HODE were elevated in patients comparing to healthy subjects also revealed higher levels in patients with invasive and bigger tumors indicating the up-regulated arachidonic acid metabolism in pituitary adenomas. Additionally, 13(S) HODE activate NF-κB and trigger pro-inflammatory responses in airway inflammation also 15-Lox-1 activity is under regulation of cytokines likewise IL-4 transcriptionally which linked the 15-Lox pathway to the immune system response and inflammation [41, 42]. Due to the role of 15-Lox pathway in the pathogenesis of several types of cancers and its direct contribution in regulating cancer cell proliferation/apoptosis, the results of the current study can provide insights in to the possible role of 15-Lox enzyme in the pathogenesis of pituitary adenoma however considering the involvement of all lipoxygenase (5-Lox, 12-Lox and 8-Lox) isoforms and their subsequent products as well as the levels of various PGs, LTs and TXs in pituitary adenoma samples is perquisite to clarify the balance status of eicosanoid cascade in pituitary adenoma pathogenesis .. Although our study provide first evidences on the 15-Lox isoforms and products status in pituitary adenoma, the arachidonic acid metabolism and 15-Lox contribution in lipid signaling and cellular functions is a complex cascade that is under regulation of many factors also can affect several mediators inside the cell, therefore characterizing the molecular mechanism and exact impact of each mediators in pituitary tumor genesis required to be worked out in connected and up-coming researches. In the current study no significant correlation was observed regarding the status of 15-Lox isoforms and products with patient’s clinic pathological features which required further comprehensive and population-based. Due to the diagnostic value of 15-Lox markers (enzymes and products) to distinguish patients from healthy subjects, the 15-Lox-1 and its product might be considered as a better potential biomarker for diagnosis of pituitary adenomas and distinguishing among hormone-secreting and non-secreting adenomas specially those adenomas with bigger tumor size and invasive features while 15-ox-2 and its product receive lower scores in its ability to distinguish different forms of pituitary adenomas.

## Conclusions

Our study provide first data regarding the expression levels of 15-Lox-1 and 15-Lox-2 as well as the circulating level of their main products in patients suffering from hormone secreting and silent pituitary adenomas. Based on our data, up regulation of both 15-Lox isoforms were observed in pituitary adenoma comparing to healthy pituitary tissues which were consistent with the elevated level of both 13(S) HODE and 15(S) HETE in patient’s serum. The higher level of 15-Lox-1 isoform and 13(S) HODE in non-hormone secreting adenomas also in acromegaly comparing to the other types of tumors were considerable which were accompanied with tumor invasion and bigger size and make 15-Lox1 a more putative biomarker for pituitary adenomas comparing to 15-Lox-2. Accordingly, the possible involvement of 15-Lipoxygense pathway with emphasize on 15-Lox-1, in the regulation of pituitary adenomas may pave the way for delineating new molecular mechanisms regarding pituitary adenoma pathogenesis and its probable therapeutic strategies.

## Data Availability

All data generated or analyzed during this study are included in this published article.

## Abbreviations

13(S)-HODE: 13-S-hydroxyoctadecadienoic acid
15(S)-HETE: 15-S-Hydroxyeicosatetraenoic acid
15-Lox: 15-Lipoxygense
AUC: Area under the curve
ETSS: Endoscopic transnasal transphenoidal surgery
FPA: Functional pituitary adenoma
HPA: Hypothalamic-pituitary-adrenal
IGF-1: Insulin-like growth factor-1
IL-4: Interleukin 4
MAP kinase: Mitogen-activated protein kinase
MMPs: Matrix-metaloproteinases
MTA1: Metastasis-associated antigen 1
NFPA: Non-functional pituitary adenomas
NF-κB: Nuclear factor kappa-light-chain-enhancer of activated B cells
PPARγ: Peroxisome proliferator-activated receptor gamma
PUFA: Poly unsaturated fatty acids
ROC: Receiver operating characteristic
ROS: Reactive oxygen species
STAT: Signal transducer and activator of transcription
